# 4-Amino-6-(piperidin-1-yl)pyrimidine-5-carbo­nitrile

**DOI:** 10.1107/S2414314620003855

**Published:** 2020-03-17

**Authors:** Radhika Bhat, K. N. Shraddha, Noor Shahina Begum

**Affiliations:** a Department of studies in Chemistry, Bangalore University, Jnana Bharathi Campus, Bangalore-560 056, Karnataka, India; University of Aberdeen, Scotland

**Keywords:** crystal structure, pyrimidine, N—H⋯N hydrogen bonds

## Abstract

In the title compound, C_10_H_13_N_5_, the piperidine ring adopts a chair conformation with the exocyclic N—C bond in an axial orientation, and the dihedral angle between the mean planes of piperidine and pyrimidine rings is 49.57 (11)°. A short intra­molecular C—H⋯N contact generates an *S*(7) ring. In the crystal, N—H⋯N hydrogen bonds link the mol­ecules into (100) sheets and a weak aromatic π-π stacking inter­action is observed [centroid–centroid separation = 3.5559 (11) Å] between inversion-related pyrimidine rings.

## Abstract 2

In the crystal, mol­ecules of the title compound are linked into (100) sheets by N—H⋯N hydrogen bonds.

## Graphical abstract

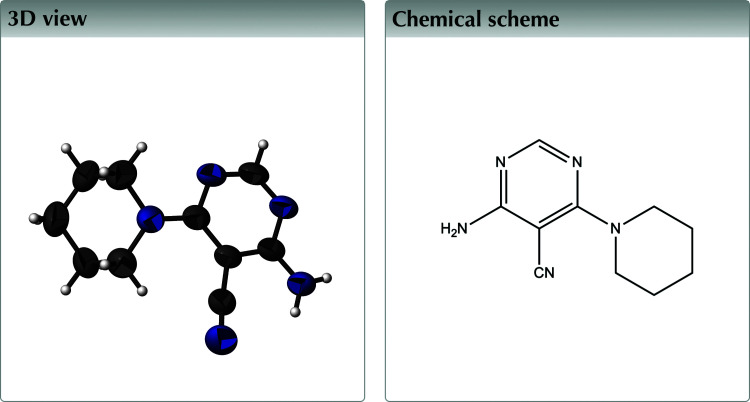

## Structure description

Pyrimidine derivatives exhibit a broad spectrum of biological activities such as GPR119 agonists (Fang *et al.*, 2019 ▸), VEGFR-2 tyrosine kinase inhibitors (Sun *et al.*, 2018 ▸) and anti­tumor activity (Hassan *et al.*, 2017 ▸). As part of our studies in this area, we now describe the synthesis and structure of the title compound.

**Figure 1:**
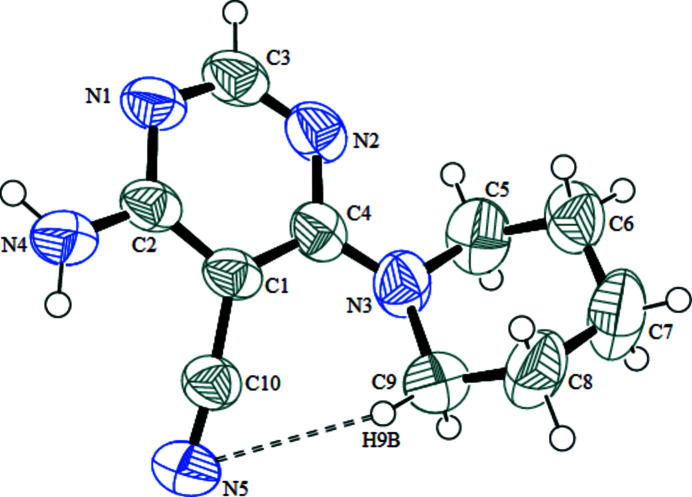
The mol­ecular structure of the title compound with displacement ellipsoids drawn at the 50% probability level. The short C—H⋯N contact is indicated by a double-dashed line.

The title compound crystallizes with one mol­ecule in the asymmetric unit (Fig. 1 ▸). The piperidine ring adopts a chair conformation, with atoms N3 and C7 displaced from the mean plane of the other four atoms (C5/C6/C8/C9) by −0.2472 (2) and 0.2133 (3) Å, respectively. The exocyclic N3—C4 bond has an axial orientation and the dihedral angle between the piperidine ring mean plane (all atoms) and the pyrimidine ring is 49.57 (11)°. A short intra­molecular C9—H9*B*⋯N5 contact generates an S(7) ring.

**Figure 2:**
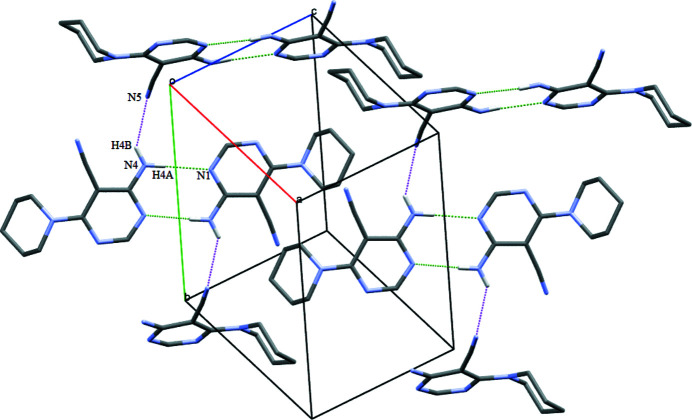
Unit-cell packing of the title compound showing N—H⋯N inter­actions as dotted green and purple lines. H atoms not involved in hydrogen bonding have been excluded.

**Figure 3:**
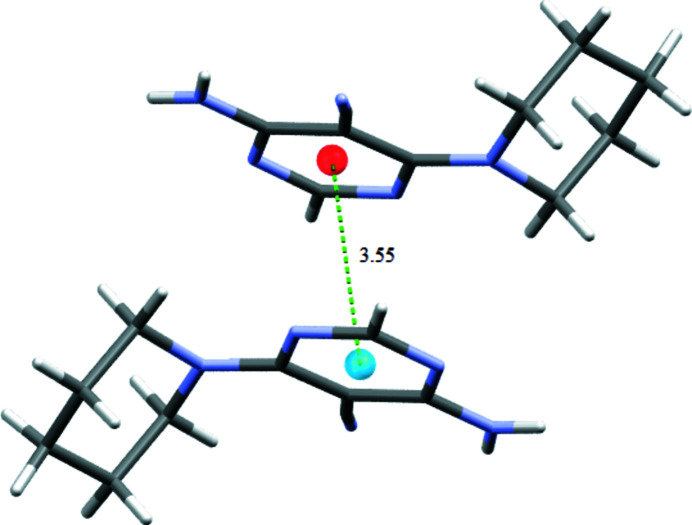
A fragment of the packing depicting the π–π inter­action as a dashed line.

In the crystal, N4—H4*A*⋯N1 hydrogen bonds (Table 1 ▸) link the mol­ecules into inversion dimers characterized by an 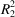 (8) graph set motif (Fig. 2 ▸) and N4—H4*B*⋯N5 hydrogen bonds link the dimers into (100) sheets. The packing also features π–π stacking inter­actions between inversion-related pyrimidine rings at a centroid–centroid distance of 3.5559 (11) Å (Fig. 3 ▸).

## Synthesis and crystallization

A mixture of 4-amino-6-chloro-pyrimidine-5-carbo­nitrile 1.0 g (0.0065 mol) and piperidine (2.75 g, 0.0325 mol) was refluxed in 20 ml of ethanol for 6 h. The reaction mixture was then cooled and stirred for 2 h at room temperature. The solid obtained was filtered, washed with ethanol and dried giving 0.98 g of white solid (yield 74%), which was recrystallized from acetone solution to obtain colourless blocks of the title compound. IR (ν, cm^−1^: 3426, 3308 (NH), 2190 (C=N), 1646 (C=N), 1223 (CN). ^1^H NMR (400 MHz DMSO-*d*_6_): δ 8.01 (*s*, 1H, pyrimidine CH), 7.21 (*br. s*, 1 N, NH_2_), 3.76 (*t*, 2H, CH_2_), 1.73–1.48 (*m*, 6H, 3CH_2_). ^13^C NMR (DMSO-*d*_6_): δ 168.5, 164.3, 159.9, 118.1, 58.9, 26.8, 24.9.

## Refinement

Crystal data, data collection and structure refinement details are summarized in Table 2 ▸.

## Supplementary Material

Crystal structure: contains datablock(s) global, I. DOI: 10.1107/S2414314620003855/hb4343sup1.cif

Structure factors: contains datablock(s) I. DOI: 10.1107/S2414314620003855/hb4343Isup2.hkl

IR. DOI: 10.1107/S2414314620003855/hb4343sup3.pdf

Proton NMR. DOI: 10.1107/S2414314620003855/hb4343sup4.pdf

C-13 NMR. DOI: 10.1107/S2414314620003855/hb4343sup5.pdf

Supporting information file. DOI: 10.1107/S2414314620003855/hb4343Isup6.cml

CCDC reference: 1988336

Additional supporting information:  crystallographic information; 3D view; checkCIF report

## Figures and Tables

**Table 1:** Hydrogen-bond geometry (Å, °)

*D*—H⋯*A*	*D*—H	H⋯*A*	*D*⋯*A*	*D*—H⋯*A*
N4—H4*A*⋯N1^i^	0.86	2.12	2.983 (2)	173
N4—H4*B*⋯N5^ii^	0.86	2.44	3.115 (3)	135
C9—H9*B*⋯N5	0.97	2.61	3.484 (1)	148

Symmetry codes: (i) 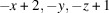 ; (ii) 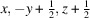 .

**Table 2:** Experimental details

Crystal data	
Chemical formula	C_10_H_13_N_5_
*M*_r_	203.25
Crystal system, space group	Monoclinic, *P*2_1_/*c*
Temperature (K)	446
*a*, *b*, *c* (Å)	10.7335 (9), 12.4005 (10), 7.9206 (6)
β (°)	93.654 (4)
*V* (Å^3^)	1052.09 (15)
*Z*	4
Radiation type	Mo *K*α
μ (mm^−1^)	0.08
Crystal size (mm)	0.18 × 0.16 × 0.15

Data collection	
Diffractometer	Bruker *SMART* *APEX* CCD
Absorption correction	Multi-scan (*SADABS*; Bruker, 1998 ▸)
No. of measured, independent and observed [*I* > 2σ(*I*)] reflections	12900, 1855, 1452
*R*_int_	0.034
(sin θ/λ)_max_ (Å^−1^)	0.595

Refinement	
*R*[*F* ^2^ > 2σ(*F* ^2^)], *wR*(*F* ^2^), *S*	0.050, 0.180, 1.16
No. of reflections	1855
No. of parameters	136
H-atom treatment	H-atom parameters constrained
Δρ_max_, Δρ_min_ (e Å^−3^)	0.21, −0.28

Computer programs: *SMART* and *SAINT* (Bruker, 1998 ▸), *SHELXS97* (Sheldrick, 2008 ▸), *SHELXL2018* (Sheldrick, 2015 ▸) and *ORTEP-3 for Windows* (Farrugia, 2012 ▸).

## full crystallographic data

### Crystal data

**Table:**

C_10_H_13_N_5_	*F*(000) = 432
*M**_r_* = 203.25	*D*_x_ = 1.283 Mg m^−^^3^
Monoclinic, *P*2_1_/*c*	Mo *K*α radiation, λ = 0.71073 Å
*a* = 10.7335 (9) Å	Cell parameters from 1855 reflections
*b* = 12.4005 (10) Å	θ = 1.9–25.0°
*c* = 7.9206 (6) Å	µ = 0.08 mm^−^^1^
β = 93.654 (4)°	*T* = 446 K
*V* = 1052.09 (15) Å^3^	Block, colourless
*Z* = 4	0.18 × 0.16 × 0.15 mm

### Data collection

**Table:**

Bruker SMART APEX CCD diffractometer	1452 reflections with *I* > 2σ(*I*)
Radiation source: fine-focus sealed tube	*R*_int_ = 0.034
ω scans	θ_max_ = 25.0°, θ_min_ = 1.9°
Absorption correction: multi-scan (SADABS; Bruker, 1998)	*h* = −12→12
	*k* = −14→14
12900 measured reflections	*l* = −9→8
1855 independent reflections	

### Refinement

**Table:**

Refinement on *F*^2^	Primary atom site location: structure-invariant direct methods
Least-squares matrix: full	Secondary atom site location: difference Fourier map
*R*[*F*^2^ > 2σ(*F*^2^)] = 0.050	Hydrogen site location: inferred from neighbouring sites
*wR*(*F*^2^) = 0.180	H-atom parameters constrained
*S* = 1.16	*w* = 1/[σ^2^(*F*_o_^2^) + (0.1087*P*)^2^ + 0.0972*P*] where *P* = (*F*_o_^2^ + 2*F*_c_^2^)/3
1855 reflections	(Δ/σ)_max_ < 0.001
136 parameters	Δρ_max_ = 0.21 e Å^−^^3^
0 restraints	Δρ_min_ = −0.28 e Å^−^^3^

### Special details

**Table:**

Geometry. All esds (except the esd in the dihedral angle between two l.s. planes) are estimated using the full covariance matrix. The cell esds are taken into account individually in the estimation of esds in distances, angles and torsion angles; correlations between esds in cell parameters are only used when they are defined by crystal symmetry. An approximate (isotropic) treatment of cell esds is used for estimating esds involving l.s. planes.
Refinement. H atoms were placed at calculated positions in the riding-model approximation, with N—H = 0.86 Å and C—H = 0.93 0.96 and 0.97 Å for aromatic, methyl and methine H atoms, respectively. The constraint *U*_iso_(H) = 1.5*U*_eq_(C) for methyl H atoms and 1.2*U*_eq_(carrier) otherwise was applied.

### Fractional atomic coordinates and isotropic or equivalent isotropic displacement parameters (Å^2^)

**Table:**

	*x*	*y*	*z*	*U*_iso_*/*U*_eq_	
C1	0.81607 (15)	0.03767 (13)	0.1080 (2)	0.0523 (5)	
C2	0.88239 (16)	0.02327 (14)	0.2655 (2)	0.0549 (5)	
C4	0.82310 (16)	−0.04487 (14)	−0.0150 (3)	0.0578 (5)	
N1	0.95761 (14)	−0.06277 (12)	0.2958 (2)	0.0637 (5)	
N3	0.75761 (15)	−0.04668 (14)	−0.1662 (2)	0.0722 (5)	
C10	0.76188 (17)	0.14099 (15)	0.0789 (2)	0.0574 (5)	
N4	0.87641 (16)	0.09400 (13)	0.3914 (2)	0.0702 (5)	
H4A	0.919123	0.083441	0.485517	0.084*	
H4B	0.829833	0.150200	0.378499	0.084*	
N2	0.90227 (15)	−0.12859 (13)	0.0178 (3)	0.0691 (5)	
N5	0.72548 (18)	0.22727 (14)	0.0646 (3)	0.0782 (6)	
C3	0.96377 (18)	−0.12964 (16)	0.1669 (3)	0.0691 (6)	
H3	1.020183	−0.186103	0.184080	0.083*	
C8	0.5344 (2)	−0.0579 (2)	−0.2263 (3)	0.0902 (8)	
H8A	0.460985	−0.016619	−0.263451	0.108*	
H8B	0.520469	−0.087903	−0.115968	0.108*	
C5	0.7794 (2)	−0.1314 (2)	−0.2914 (3)	0.0855 (7)	
H5A	0.854903	−0.170765	−0.256953	0.103*	
H5B	0.790807	−0.098612	−0.400522	0.103*	
C6	0.6712 (2)	−0.2074 (2)	−0.3063 (3)	0.0802 (7)	
H6A	0.665402	−0.245504	−0.200085	0.096*	
H6B	0.684841	−0.260338	−0.393412	0.096*	
C9	0.6452 (2)	0.01582 (19)	−0.2108 (3)	0.0776 (6)	
H9A	0.653908	0.052850	−0.317313	0.093*	
H9B	0.633297	0.069592	−0.124387	0.093*	
C7	0.5513 (3)	−0.1489 (3)	−0.3494 (4)	0.1092 (10)	
H7A	0.481912	−0.198846	−0.345855	0.131*	
H7B	0.551740	−0.120234	−0.463297	0.131*	

### Atomic displacement parameters (Å^2^)

**Table:**

	*U*^11^	*U*^22^	*U*^33^	*U*^12^	*U*^13^	*U*^23^
C1	0.0429 (9)	0.0413 (9)	0.0733 (12)	−0.0013 (7)	0.0095 (8)	0.0058 (8)
C2	0.0461 (9)	0.0421 (9)	0.0771 (13)	−0.0017 (7)	0.0087 (8)	0.0062 (8)
C4	0.0398 (9)	0.0528 (10)	0.0819 (13)	−0.0079 (7)	0.0118 (8)	−0.0037 (9)
N1	0.0569 (10)	0.0484 (9)	0.0858 (12)	0.0083 (7)	0.0045 (8)	0.0039 (8)
N3	0.0522 (10)	0.0749 (12)	0.0894 (13)	−0.0009 (8)	0.0024 (8)	−0.0172 (9)
C10	0.0547 (10)	0.0478 (11)	0.0695 (12)	−0.0035 (8)	0.0036 (8)	0.0065 (8)
N4	0.0774 (11)	0.0569 (10)	0.0753 (12)	0.0180 (8)	−0.0036 (8)	−0.0008 (8)
N2	0.0502 (9)	0.0555 (10)	0.1022 (14)	0.0033 (7)	0.0089 (9)	−0.0119 (9)
N5	0.0879 (13)	0.0496 (10)	0.0955 (15)	0.0058 (9)	−0.0071 (10)	0.0081 (8)
C3	0.0503 (11)	0.0509 (11)	0.1066 (17)	0.0086 (8)	0.0076 (10)	−0.0009 (11)
C8	0.0557 (13)	0.118 (2)	0.0950 (17)	0.0059 (12)	−0.0105 (11)	−0.0233 (15)
C5	0.0727 (14)	0.1028 (19)	0.0825 (16)	0.0020 (13)	0.0154 (11)	−0.0248 (14)
C6	0.0959 (17)	0.0833 (15)	0.0607 (13)	−0.0066 (13)	0.0000 (11)	−0.0158 (10)
C9	0.0793 (15)	0.0737 (14)	0.0787 (15)	0.0076 (11)	−0.0039 (11)	0.0027 (11)
C7	0.0790 (17)	0.137 (3)	0.109 (2)	−0.0090 (16)	−0.0162 (14)	−0.0462 (19)

### Geometric parameters (Å, º)

**Table:**

C1—C2	1.408 (3)	C8—C9	1.499 (3)
C1—C4	1.418 (3)	C8—C7	1.510 (4)
C1—C10	1.420 (3)	C8—H8A	0.9700
C2—N4	1.332 (2)	C8—H8B	0.9700
C2—N1	1.350 (2)	C5—C6	1.495 (3)
C4—N3	1.350 (3)	C5—H5A	0.9700
C4—N2	1.356 (3)	C5—H5B	0.9700
N1—C3	1.321 (3)	C6—C7	1.498 (4)
N3—C9	1.459 (3)	C6—H6A	0.9700
N3—C5	1.474 (3)	C6—H6B	0.9700
C10—N5	1.142 (2)	C9—H9A	0.9700
N4—H4A	0.8600	C9—H9B	0.9700
N4—H4B	0.8600	C7—H7A	0.9700
N2—C3	1.316 (3)	C7—H7B	0.9700
C3—H3	0.9300		
			
C2—C1—C4	118.10 (16)	H8A—C8—H8B	107.8
C2—C1—C10	115.91 (16)	N3—C5—C6	110.29 (18)
C4—C1—C10	125.39 (18)	N3—C5—H5A	109.6
N4—C2—N1	116.35 (18)	C6—C5—H5A	109.6
N4—C2—C1	122.27 (16)	N3—C5—H5B	109.6
N1—C2—C1	121.37 (17)	C6—C5—H5B	109.6
N3—C4—N2	116.21 (18)	H5A—C5—H5B	108.1
N3—C4—C1	125.02 (18)	C5—C6—C7	111.4 (2)
N2—C4—C1	118.76 (19)	C5—C6—H6A	109.4
C3—N1—C2	114.68 (18)	C7—C6—H6A	109.4
C4—N3—C9	125.62 (18)	C5—C6—H6B	109.4
C4—N3—C5	120.85 (19)	C7—C6—H6B	109.4
C9—N3—C5	112.34 (18)	H6A—C6—H6B	108.0
N5—C10—C1	174.5 (2)	N3—C9—C8	109.6 (2)
C2—N4—H4A	120.0	N3—C9—H9A	109.8
C2—N4—H4B	120.0	C8—C9—H9A	109.8
H4A—N4—H4B	120.0	N3—C9—H9B	109.8
C3—N2—C4	116.86 (17)	C8—C9—H9B	109.8
N2—C3—N1	129.86 (18)	H9A—C9—H9B	108.2
N2—C3—H3	115.1	C6—C7—C8	110.6 (2)
N1—C3—H3	115.1	C6—C7—H7A	109.5
C9—C8—C7	112.4 (2)	C8—C7—H7A	109.5
C9—C8—H8A	109.1	C6—C7—H7B	109.5
C7—C8—H8A	109.1	C8—C7—H7B	109.5
C9—C8—H8B	109.1	H7A—C7—H7B	108.1
C7—C8—H8B	109.1		
			
C4—C1—C2—N4	−176.67 (15)	C1—C4—N3—C5	174.72 (17)
C10—C1—C2—N4	11.7 (2)	N3—C4—N2—C3	−177.91 (17)
C4—C1—C2—N1	4.5 (3)	C1—C4—N2—C3	3.0 (3)
C10—C1—C2—N1	−167.09 (16)	C4—N2—C3—N1	2.8 (3)
C2—C1—C4—N3	174.67 (16)	C2—N1—C3—N2	−4.6 (3)
C10—C1—C4—N3	−14.6 (3)	C4—N3—C5—C6	108.9 (2)
C2—C1—C4—N2	−6.3 (3)	C9—N3—C5—C6	−59.3 (3)
C10—C1—C4—N2	164.43 (16)	N3—C5—C6—C7	55.9 (3)
N4—C2—N1—C3	−178.31 (16)	C4—N3—C9—C8	−109.3 (2)
C1—C2—N1—C3	0.6 (3)	C5—N3—C9—C8	58.3 (3)
N2—C4—N3—C9	162.23 (19)	C7—C8—C9—N3	−55.0 (3)
C1—C4—N3—C9	−18.7 (3)	C5—C6—C7—C8	−52.9 (3)
N2—C4—N3—C5	−4.3 (3)	C9—C8—C7—C6	52.8 (3)

### Hydrogen-bond geometry (Å, º)

**Table:**

*D*—H···*A*	*D*—H	H···*A*	*D*···*A*	*D*—H···*A*
N4—H4*A*···N1^i^	0.86	2.12	2.983 (2)	173
N4—H4*B*···N5^ii^	0.86	2.44	3.115 (3)	135
C9—H9*B*···N5	0.97	2.61	3.484 (1)	148

Symmetry codes: (i) −*x*+2, −*y*, −*z*+1; (ii) *x*, −*y*+1/2, *z*+1/2.
