# Supplementary figures and images for: 4-Amino-6-(piperidin-1-yl)pyrimidine-5-carbo­nitrile
Source: IUCrdata. 2020 Mar 17;5(Pt 3):x200385. doi: 10.1107/S2414314620003855 (PMC9462203; doi:10.1107/S2414314620003855)

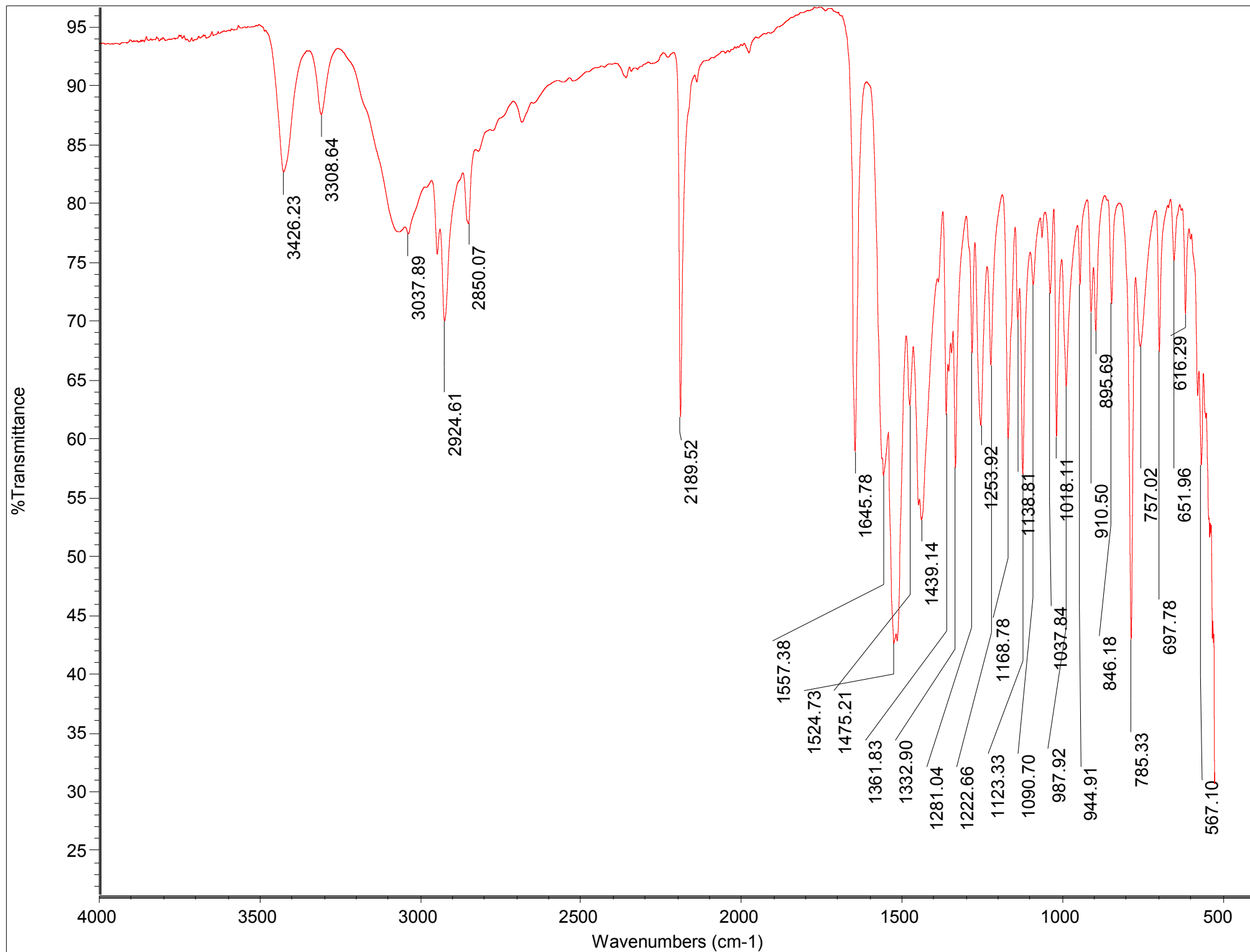

Supplement: Supplementary file 3 [file x-05-x200385-sup3.pdf]

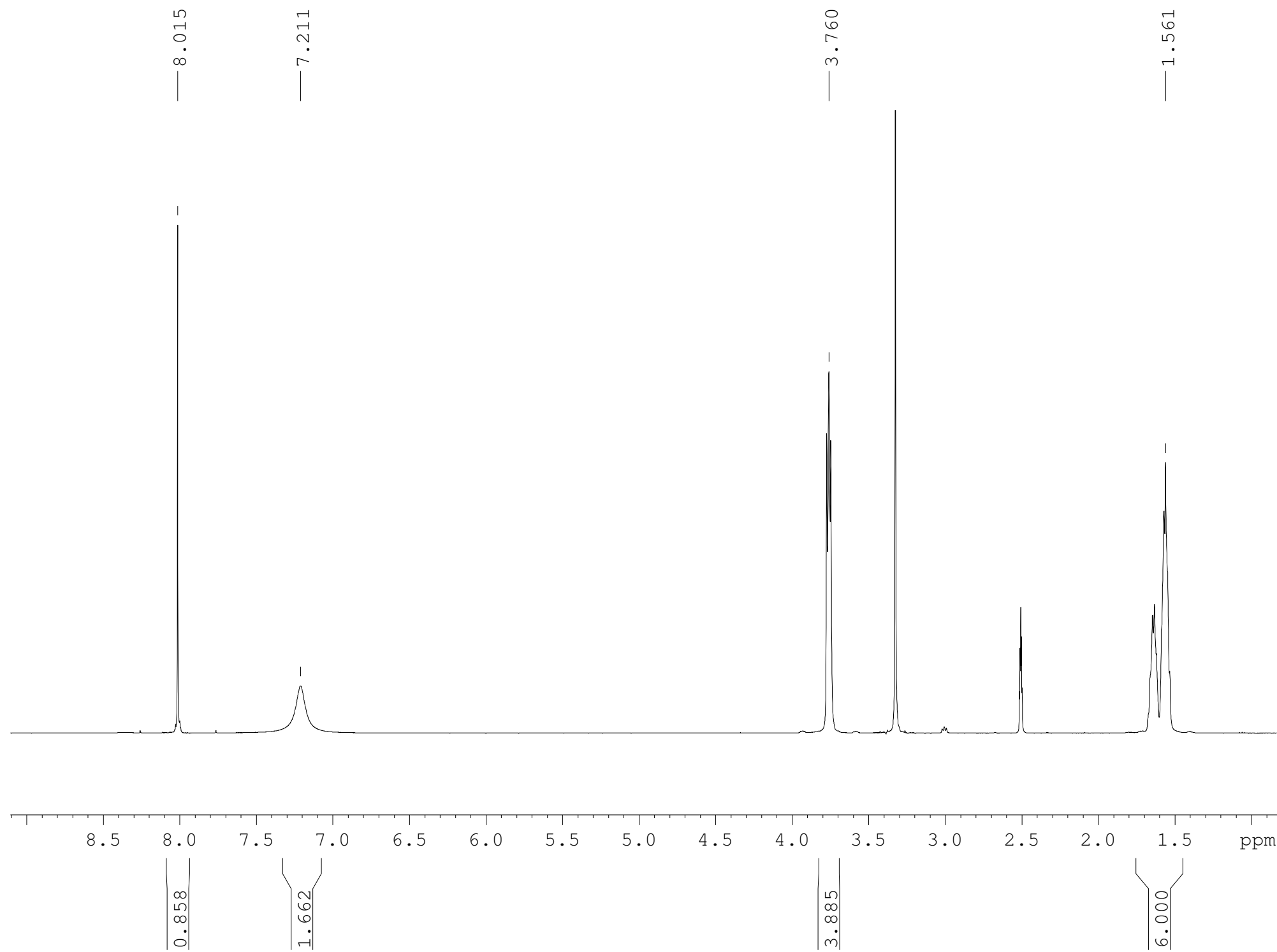

Supplement: Supplementary file 4 [file x-05-x200385-sup4.pdf]

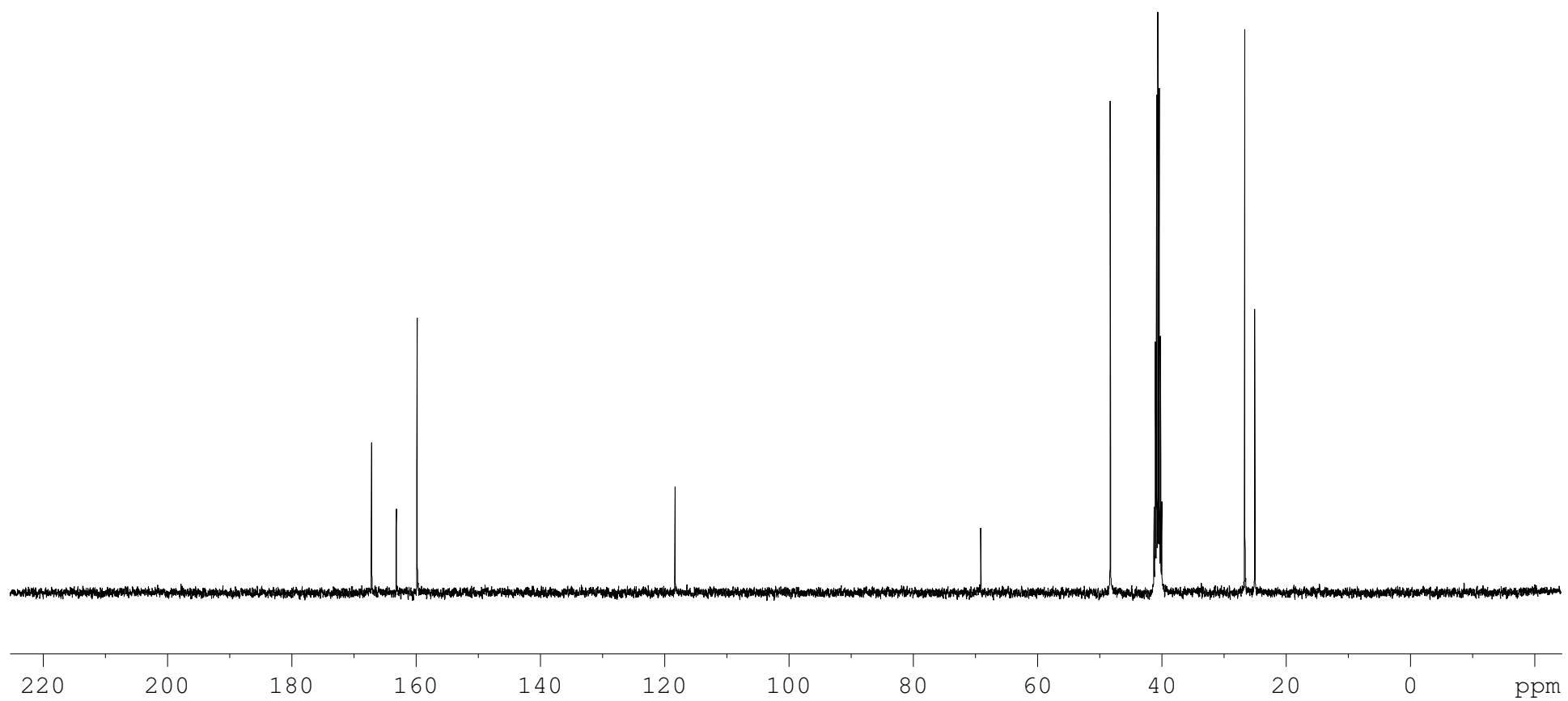

Supplement: Supplementary file 5 [file x-05-x200385-sup5.pdf]
